# Evidence-Based Exercise Recommendations for Older Adults With Cognitive Impairments

**DOI:** 10.1093/geroni/igaa057.3116

**Published:** 2020-12-16

**Authors:** Patricia Heyn, Alex Tagawa, Ahmed Negm, Pallavi Sood

**Affiliations:** 1 University of Colorado Anschutz Medical Campus, Aurora, Colorado, United States; 2 Children’s Hospital Colorado, Aurora, Colorado, United States; 3 University of Alberta, Edmonton, Alberta, Canada; 4 University of Florida, Gainesville, Florida, United States

## Abstract

Since the publishing of our meta-analysis evaluating the effects of randomized exercise trials on cognitive function of Older Adults with Cognitive Impairments (OAwCIs) (Heyn et al 2004), several meta-analysis reviews were published addressing similar question. We currently appraised this evidence and preliminary synthesis of twelve, well-designed meta-analysis reports resulted in 193 RCTs and 15,614 participants over the age of 65 years old diagnosed with MCI or Alzheimer’s disease (AD). Exercise prescription paradigms averaged 156 minutes per week for 20-week. The combined cognitive function outcome mean effect size was medium; 0.67 (0.06-1.34 95% CI). Grounded in this unique umbrella study results, sustained and prolonged exercise training might provide an effective intervention for the maintenance or enhancement of cognitive function for MCI and AD. This comprehensive meta-analysis umbrella offers valuable and strong exercise recommendations for OAwCIs. This study results will be of great significance to professionals involved in the care of OAwCIs.

